# Quantitative electroencephalography as a potential neurophysiological diagnostic biomarker of schizophrenia and first-episode psychosis: a systematic review of clinical implications

**DOI:** 10.3389/fpsyt.2026.1823040

**Published:** 2026-06-12

**Authors:** Kacper Łoś, Napoleon Waszkiewicz

**Affiliations:** Department of Psychiatry, Medical University of Bialystok, Bialystok, Poland

**Keywords:** biomarker, electroencephalography, first-episode psychosis, psychosis, qEEG, quantitative electroencephalography, schizophrenia

## Abstract

**Background and Hypothesis:**

Schizophrenia affects approximately 1% of the global population, with early diagnosis critical for optimal treatment outcomes. Resting-state quantitative electroencephalography (qEEG) represents a promising, non-invasive biomarker. We hypothesized that 18 years after the seminal Boutros review, advances in qEEG methodology would demonstrate characteristic neurophysiological signatures distinguishing schizophrenia from healthy controls and first-episode psychosis.

**Study Design:**

This systematic review followed PRISMA guidelines, searching PubMed, Google Scholar, and Scopus for studies published after 2008. Inclusion criteria required adult human studies comparing resting-state qEEG in patients with schizophrenia or first-episode psychosis versus healthy controls. Studies employing advanced artificial intelligence techniques, evoked potentials, and task-based recordings were excluded to focus on classical qEEG parameters applicable in clinical settings from 467 publications after temporal restriction, 19 original studies met inclusion criteria. Across the studies included in this review, a total of 1242 patients were analyzed, comprising 981 individuals diagnosed with schizophrenia and 261 individuals with a first episode of psychosis. These cohorts were compared with 1211 healthy control participants across studies.

**Study Results:**

Chronic schizophrenia patients consistently demonstrated increased delta and theta wave activity in anterior regions and decreased alpha peak frequency in posterior areas. These alterations were less pronounced or absent in first-episode psychosis, suggesting progression with disease duration or long-term treatment. The novel theta/alpha component (6–9 Hz) identified by Nakhnikian et al. provides mechanistic insight into alpha slowing. Significant methodological heterogeneity precluded meta-analysis.

**Conclusions:**

Characteristic qEEG alterations exist in chronic schizophrenia but remain inconsistent in early psychosis. The unique theta/alpha signature represents a promising specific biomarker. However, persistent methodological heterogeneity limits clinical translation. Standardized multicenter protocols with longitudinal designs are essential before qEEG implementation in routine schizophrenia diagnosis.

## 1 Introduction

Electroencephalography (EEG), a non-invasive method of recording electrical cortical activity via electrodes placed on the scalp surface, has been widely used in neurological diagnostics for years (1, 2). It also constitutes an integral element of psychiatric diagnostics (3, 4). In neuropsychiatry, EEG finds application primarily in the differential diagnosis of psychotic disorders, enabling differentiation of first-episode psychosis from organic conditions such as non-convulsive status epilepticus (5, 6). The fundamental and crucial element of EEG analysis is assessment of background activity, characterized in healthy individuals by dominant alpha waves (8–12 Hz) recorded in posterior regions (7). Assessment also includes focal and generalized changes and epileptiform discharges, whose presence has the greatest clinical significance in organic central nervous system disorders (8). As early as the 1970s, it was noted that in patients with schizophrenia, alpha activity is diminished, with increased beta and theta waves activity (7). However, traditional EEG analysis relies on subjective assessment of these patterns, which constitutes a significant limitation of the method (9). The development of digital technologies enabled quantitative EEG analysis (qEEG), mainly through application of fourier transformation, opening new possibilities in assessing neurophysiological aspects of diagnosis and differentiation of disorders (10, 11). First attempts at quantitative EEG analysis date back to the 1940s (7); however, systematic development of qEEG began in the 1970s with the work of E. Roy John and colleagues, and the formal concept of qEEG was later defined by Mark Nuwer in 1997 (12, 13).

Schizophrenia is a serious psychiatric disorder affecting approximately 1% of the global population, characterized by disturbances in thinking, perception, emotions, speech, and behavior (14). The disease typically has a chronic course with periods of exacerbation and remission (15). Its clinical heterogeneity may cause diagnostic difficulties, particularly in early stages when symptoms may be non-specific or resemble other psychiatric disorders (16, 17). Furthermore, current diagnostic criteria are based mainly on clinical symptoms and subjective assessment, which may lead to delays in diagnosis and initiation of appropriate treatment (15, 18). The pathophysiological process of schizophrenia, like many neurodegenerative diseases, probably begins long before the onset of clinical symptoms (19, 20). It may be preceded by prodromal negative symptoms, which emphasizes the importance of early recognition (21). Schizophrenia has a negative impact on patients’ lives, often leading to social exclusion and burden for families (22, 23). For this reason, the search for reliable, inexpensive, and clinically applicable biomarkers of schizophrenia is extremely important (24, 25). The discovery of such a marker could enable future development of drugs with causal or disease-modifying effects (26, 27). Currently used antipsychotic medications are introduced typically at disease diagnosis and have only symptomatic effects (28). Although research on biomarkers in blood, urine, saliva, and genetic markers continues, resting-state qEEG constitutes a promising method due to its non-invasiveness, short examination duration, relatively low cost, and minimal involvement of medical staff (29, 30).

Worldwide, research is also being conducted on neurobiomarkers using advanced neuroimaging techniques however, these methods are expensive, time-consuming, and require specialized equipment, which currently limits their widespread application in routine clinical practice (31). In this context, qEEG emerges as an easily accessible method combining high temporal resolution with relatively low costs and availability (32). In 2008, a literature review by Boutros and colleagues was published, evaluating resting-state quantitative EEG (qEEG) as a potential biomarker for schizophrenia (5). This review, including earlier studies, revealed reproducible patterns of EEG spectral changes in patients with schizophrenia, such as increased slow wave activity (delta and theta) and decreased alpha wave activity (5). However, the limited number of multicenter studies using standardized protocols and inconsistent data analysis methods hindered clear interpretation of results and limited conclusions about the clinical utility of qEEG. The aim of this article is to systematically analyze literature on the application of resting-state quantitative EEG as a potential biomarker for schizophrenia, published after 2008. This review will consider advances in qEEG data analysis methodology, assess progress toward standardizing research methodology and evaluate the clinical potential of qEEG. This analysis will allow verification of research directions proposed in the 2008 review and determination of the current state of knowledge in this field.

## 2 Materials and methods

### 2.1 Study design and registration

This systematic review was conducted according to PRISMA (Preferred Reporting Items for Systematic Reviews and Meta-Analyses) guidelines (33).

### 2.2 Search strategy

Literature search in PubMed, Google Scholar, and Scopus databases was performed manually by two independent researchers. The following terms were used with Boolean operators: “quantitative electroencephalography’’, “schizophrenia”, “qEEG’’, “spectral electroencephalography’’, “psychosis”, “irst psychotic episode’’, limiting results to publications in English and published after 2008. An example search query structure was as follows:

“quantitative electroencephalography’’ OR “qEEG’’ OR “spectral electroencephalography’’ AND “schizophrenia’’ OR “psychosis’’ OR “first-psychotic episode’’.

### 2.3 Inclusion and exclusion criteria

The inclusion and exclusion criteria for studies included in the systematic review are presented in **Table 1**.

**Table 1:** Inclusion and exclusion criteria for the review.

Criterion	Inclusion	Exclusion
Publication date	Studies published after 2008	Studies published before 2008
Included articles	Only peer-reviewed	Not peer-reviewed
Study group	Adults; Comparison of patients with schizophrenia (or first-episode psychosis) with healthy control group	Children; No healthy control group or no group of patients with schizophrenia/first-episode psychosis
Language	Publications in English	Publications in other languages
Study type	Human studies	Animal studies, *in vitro* studies
Publication type	Original research papers (scientific articles), Literature reviews, meta-analyses	Case reports, expert opinions
EEG method	Quantitative/spectral EEG analysis at rest	Evoked potentials,
EEG conditions	Resting-state EEG	Task-based EEG
Data analysis methods	classical statistical analysis	artificial intelligence (AI)
Study objective	Quantitative assessment of EEG spectra in schizophrenia compared to healthy population	Treatment efficacy assessment only, treatment response prediction

The review excluded studies of evoked potentials, task-based qEEG recordings, and artificial intelligence (AI) applications, as well as studies evaluating only treatment efficacy or treatment response prediction. AI approaches show promising results in qEEG analysis, their complex methodology and current limitations in clinical implementation warrant their exclusion from this review focused on classical qEEG parameters.

### 2.4 Study selection

Search using the keywords initially returned 467 results. After adding a time restriction (publications from 2008), this number decreased to 341. Subsequently, 328 records were excluded after abstract review based on pre-defined exclusion criteria. Initially, 8 articles meeting the predefined inclusion criteria were identified through the primary database search. Subsequently, an additional 11 studies were identified through manual screening of the reference lists of the included articles, in which relevant resting-state EEG data eligible for inclusion in this systematic review were found. Ultimately, after analyzing abstracts and full texts, 19 original papers were included in the review.

**Figure 1:**
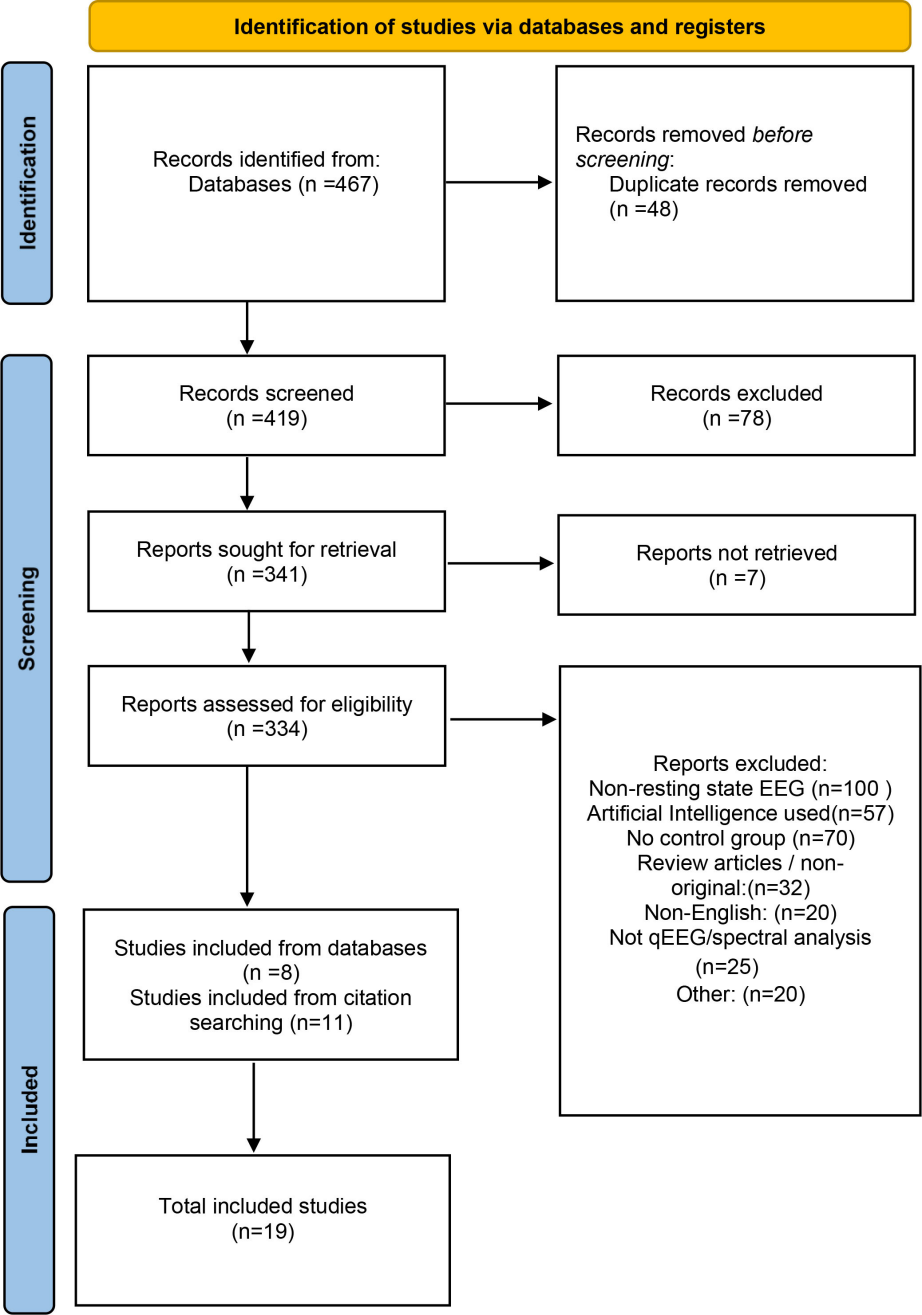
PRISMA checklist.

The study selection process is presented in **Figure 1**.

### 2.5 Data extraction and analysis

From selected papers, data regarding characteristic EEG spectral features in patients with schizophrenia compared to control groups were collected. Collected data included parameters such as dominant wave frequency, wave amplitudes, and brain activity topography. Due to the heterogeneity of data analysis methods in selected studies, quantitative meta-analysis was not performed. Results are presented as a discussion of data regarding individual qEEG bands (delta, theta, alpha, beta, gamma) considering similarities and differences between individual studies.

## 3 Results

The systematic literature search and study selection process followed PRISMA guidelines, as illustrated in **Figure 1** (33). Results are discussed by individual frequency bands (from lowest to highest frequencies). The results of studies included in the review are presented in **Table 2**. Across the studies included in this review, a total of 1242 patients were analyzed, comprising 981 individuals diagnosed with schizophrenia and 261 individuals with a first episode of psychosis. These cohorts were compared with 1211 healthy control participants across studies. A comparison of the results included in the review in terms of qEEG changes described in first-episode psychosis and in chronic schizophrenia is presented in **Table 3**.

**Table 2:** Characteristics of included original studies.

Author, year	Study group	Control group	Number of electrodes	Analyzed bands	Main findings
Begić et al., 2011 (34)	Schizophrenia n=30	n=30	19	Delta (0.5-4), Theta (4-8), Alpha (8-13), Beta (13-30)	Increased delta, theta, and beta power; decreased alpha power
Ranlund et al., 2014 (35)	Schizophrenia n=48, First episode n=87	n=107	19	Delta (1.95-3.90), Theta (4.39-7.32), Alpha (7.81-12.7), Beta (13.2-21)	Higher delta and theta amplitude only in patients with chronic schizophrenia
Kim et al., 2015 (36)	Schizophrenia n=90	n=90	19	Delta (1-4), Theta (4-8), Alpha1 (8-10), Alpha2 (10-12), Beta (12-25)	Increased delta and theta activity, decreased alpha2
Yeum et al., 2018 (37)	Schizophrenia n=31	n=31	19	Alpha (8-13)	Lower alpha peak frequency and coherence on quantitative electroencephalography
Renaldi et al., 2019 (38)	First-episode psychosis n=24	n=24	21	Delta (0.5-4)	Higher delta power in anterior and posterior regions in patients with first-psychosis episode
Yadav et al., 2021 (39)	First-episode psychosis n=29	n=30	19	Gamma (31-100)	Higher gamma spectral power (31–50 Hz and 51–70 Hz) in patients
Dominicus et al., 2023 (40)	First-episode psychosis n=62	n=102	32	Delta (0.5-4), Theta (4-8), Alpha (8-13), Beta (13-30)	No significant differences after multiple testing correction
Nakhnikian et al., 2024 (41)	Schizophrenia n=39	n=36	Dense electro dearray	Whole spectrum (0.5-30)	Decreased alpha peak frequency, presence of unique component in theta/alpha range (6–9 Hz)
Tikka et al. (2014) (42)	First-episode psychosis n=37	n=30	192	Gamma: low gamma (30–50 Hz), high gamma 1 (51–70 Hz), high gamma 2 (71–100 Hz)	significantly higher low gamma power (30–50 Hz) in the left frontal and left parietal regions
Goldstein et al. (2015) (43)	Schizophrenia n=13	n=13	256	alpha (8–12 Hz)	reduced alpha-band power particularly in frontal and occipital regions
Murphy et al (2019) (44)	First-episode psychosis n=22	n=22	128	alpha (8–12 Hz)	significantly reduced peak alpha frequency
Kam et al. (2014) (45)	Schizophrenia n=132	n=136	32	Delta (0.5–4 Hz), Theta (4–8 Hz), Alpha1 (8–10 Hz), Alpha2 (10–12 Hz), Beta1 (12–20 Hz), Beta2 (20–30 Hz), Gamma (30–50 Hz)	increased intrahemispheric delta coherence, increased intrahemispheric alpha1/alpha2 coherence in selected regions, and increased interhemispheric temporal alpha1 and alpha2 coherence
Hanslmay et al. (2012) (46)	Schizophrenia n=26	n=26	61	delta (1–3 Hz), theta (4–7.5 Hz), alpha (8–11.5 Hz), beta1 (12–19.5 Hz), beta2 (20–29.5 Hz), gamma (30–44.5 Hz)	significantly higher theta power than healthy controls; the difference was mainly in the fronto-central and right parietal regions
Hong et al. (2012) (47)	Schizophrenia n= 128	n=110	28	delta (1–4 Hz), theta-alpha (5–11 Hz), beta (12–20 Hz), low gamma (20–40 Hz), gamma (40–85 Hz), high gamma (>85 Hz)	significantly increased oscillatory energy, the most pronounced difference was in the theta-alpha range of 5–11 Hz; an increase in gamma (40–85 Hz) and delta (1–4 Hz) was observed in patients relative to controls
Bandyopadhyaya et al. (2011) (48)	Schizophrenia n=20	n=20	32	Gamma (30-100Hz)	patients with schizophrenia showed significantly lower interhemispheric spontaneous gamma coherence and increased gamma power compared to healthy controls
Mitra et al. (2017) (49)	Schizophrenia n=15	n=15	192	alpha, theta	significantly lower baseline alpha/theta ratio (ATR) in the left frontal and temporal regions
Cecchi et al. (2023) (50)	Schizophrenia n=80	n=81	7	delta, theta, alpha1, alpha2, beta1, beta2, beta3, gamma	higher absolute delta power, lower relative beta1 and beta2 power and higher theta/beta ratio compared to healthy volunteers;
Ramsey et al. (2021) (51)	Schizophrenia n=104	n=108	62	Alpha (7,25–13 Hz)	Reduced individual alpha peak frequency (IAPF) across all electrode sites; mean IAPF 9.41 Hz in schizophrenia vs 9.91 Hz in healthy controls
Narayanan et al. (2014) (52)	Schizophrenia n= 225	n= 200	64	Delta, Theta, Alpha (slow/fast), Beta (slow/fast)	Increased delta, theta, slow alpha, and fast alpha activity, as well as increased fronto-central slow beta activity in schizophrenia compared to healthy controls No differences in fast-beta band

**Table 3:** Comparison of qEEG changes between first-episode psychosis and chronic schizophrenia based on included studies.

Frequency band	First-episode psychosis	Chronic schizophrenia
Delta (0.5-4Hz)	Renaldi (38): ↑ absolute power in anterior and posterior regions	Begić (34): ↑ absolute power (multi-regional)
	Dominicus (40): no significant differences after multiple testing correction	Ranlund (35): ↑ amplitude in patients with chronic psychosis
	Redwan (25): no significant differences in broad bands	Kim (36): ↑ activity, particularly fronto-central
Theta (4–8 Hz)	Ranlund (35): no significant differences in first-episode patients	Begić (34): ↑ absolute power (Fp1, Fp2, F3, F4, F7, T3, T4)
		Ranlund (35): ↑ amplitude only in patients with chronic psychosis
		Kim (36): ↑ fronto-central activity
		Nakhnikian (41): presence of unique theta/alpha component (6–9 Hz)
		Mitra ( (49): lower baseline alpha/theta ratio (ATR) in the left frontal and temporal regions
Alpha (8–13 Hz)	Renaldi (38): no significant differences in absolute power	Yeum (37): ↓ peak frequency in occipital region
	Redwan (61): no differences in broad bands, differences in narrow intervals (10.25-11.25 Hz)	
		Begić (34): ↓ absolute power (regional)
		Kim (36): ↓ alpha2 activity
		Nakhnikian (41): ↓ peak frequency
Beta (13-30Hz)	Renaldi (38): no significant differences	Begić (34): ↑ absolute power in frontal and temporal regions
	Dominicus (40): no significant differences after correction	Kim (36): no significant differences
Gamma (>30Hz)	Yadav (39): ↑ spectral power (31–50 Hz and 51–70 Hz)	No data in analyzed studies

**Table 3** Comparison of qEEG changes between first-episode psychosis and chronic schizophrenia based on included studies.

For this review, we operationally define “chronic schizophrenia” as patients with an established diagnosis of schizophrenia with disease duration typically exceeding 2 years, while “first-episode psychosis” refers to patients experiencing their first psychotic episode, regardless of final diagnostic outcome.

### 3.1 Delta frequency (0.5–4 Hz)

The delta band represents the slowest brain waves recorded in electroencephalography, characterized by waves with the highest amplitude among all bands (53). It is generated mainly by deep subcortical brain structures and plays an important role in processes related to deep sleep and body regeneration (54). Delta activity is most prominent during deep sleep (stages 3 and 4 of NREM sleep), relaxation, and meditation. In infants and young children, delta activity is significantly greater than in adults, which is related to intensive central nervous system development (55). In clinical practice, it should be remembered that delta activity, especially in frontal regions, requires differentiation from eye movement artifacts. The 2008 review indicated increased proportion of delta band in patients with schizophrenia compared to healthy groups (5). In the Renaldi et al. (2019) study, it was evaluated whether total resting EEG power could predict symptom improvement and functioning in patients with first-episode psychosis after one year of treatment (38). Patients with first-episode psychosis (n=24) showed significantly higher absolute delta wave power in anterior and posterior regions compared to the control group (n=24). This study also found that higher delta power in the posterior region was a positive predictor of positive symptom improvement and general functioning after one year. Lower delta power in the anterior region predicted improvement in negative symptoms and general functioning after one year. Increased absolute delta wave power may be associated with the onset of psychotic disorders. Decreased delta power in anterior regions and increased in posterior regions may serve as predictive markers of better prognosis in patients with first-episode psychosis, which may support early clinical intervention. The authors emphasize that the influence of antipsychotic treatment on analysis results cannot be excluded. The Begić et al. (2011) study, including 30 patients with schizophrenia, 30 patients with depression (depression patients were not analyzed in the current review), and 30 healthy individuals, showed increased absolute delta wave power in patients with schizophrenia compared to the control group (statistically significant in many brain regions) (34). Differences between the schizophrenia and depression groups were statistically significant in prefrontal regions (Fp1, Fp2, F4, F8). Ranlund et al. (2014) investigated whether resting-state qEEG could serve as an endophenotype of psychosis, including whether it could indicate genetic predisposition to this disorder (35). In the delta band (1.95-3.90 Hz), patients with schizophrenia (n=48) showed significantly higher delta wave amplitude compared to the control group (n=107). In other groups (patients with first-episode psychosis, at-risk individuals, healthy relatives of patients), no significant differences were found. Possible influence of pharmacological treatment on results was not completely excluded by the authors. Kim et al. (2015) observed increased delta wave activity in patients with schizophrenia (n=90) compared to the control group (n=90), especially in the fronto-central region (36). The best results in ROC analysis were obtained for the delta band (classification accuracy 62.2%). In the manuscript by Dominicus et al. (2023), absolute delta wave power was analyzed in patients with first-episode psychosis (n=62) compared to healthy control subjects (n=102) (40). After multiple testing correction, no significant differences were found in main parameters of delta, theta, alpha, and beta bands between the study and control groups. It is worth noting that before correction, some differences were found (e.g., higher relative delta power in patients with first-episode psychosis). Kam et al. investigated resting-state EEG in schizophrenia, bipolar disorder, and non-psychiatric controls. In schizophrenia, no significant differences in delta or theta power were found relative to controls, whereas increased intrahemispheric delta coherence and increased intra- and interhemispheric alpha coherence were observed in selected regions (45). Analysis of narrow frequency bands within the delta band also showed differences. These subtle differences, which may be hidden by averaging data in broader bands, indicate the probable need for very precise EEG data analysis, suggesting that more advanced EEG analysis methods may be useful in identifying schizophrenia biomarkers.

### 3.2 Theta frequency (4–8 Hz)

The theta band is a frequency range of brain waves associated with various mental states and cognitive processes (56). Theta activity usually has greater amplitude than beta and gamma waves (11). The source of theta waves is mainly subcortical structures such as the hippocampus and parahippocampal gyrus, which play an important role in memory processes and learning (57). Theta activity is particularly important in the context of cognitive processes related to spatial memory, navigation, associative learning, and integration of information from various sources. According to the 2008 review, an increase in theta wave proportion is expected in patients with schizophrenia compared to the healthy population (5). The Begić et al. (2011) study showed increased absolute theta wave power in regions Fp1, Fp2, F3, F4, F7, T3, and T4 in patients with schizophrenia compared to the control group (34). Similar conclusions were presented by Kim et al. (2015), where increased theta wave activity was observed in patients with schizophrenia compared to the control group, especially in the fronto-central region (36). The Ranlund et al. (2014) study showed that only in patients with chronic psychosis was significantly higher theta wave amplitude (4.39-7.32 Hz) observed compared to the control group and patients with first-episode psychosis and relatives of ill patients (35). This result suggests that changes in theta activity may be related to disease duration or long-term use of antipsychotic medications. Nakhnikian et al. (2024) using principal component analysis demonstrated the presence of a unique spectral component in the theta/alpha range (6–9 Hz), characteristic only for patients with schizophrenia (41). This unique oscillation was not found in healthy controls and may represent a specific neurophysiological signature of schizophrenia. The source distribution of this unique theta/alpha component involved mainly prefrontal and parahippocampal areas. Cecchi et al. (2023) evaluated a standardized suite of ERP and QEEG biomarkers in patients with schizophrenia and healthy volunteers within a multicenter validation study. In the resting-state EEG component, schizophrenia subjects showed increased absolute delta power, reduced relative beta1 and beta2 power, and an increased theta/beta ratio compared with controls (50). Hanslmayr et al. showed that patients with schizophrenia exhibited increased theta power during resting-state EEG compared with healthy controls, particularly over fronto-central and right parietal regions (46). Hong et al. demonstrated that patients with schizophrenia showed broadly increased oscillatory energy in resting EEG, with the most prominent abnormality in the theta-alpha range (5–11 Hz) (47).

### 3.3 Alpha frequency (8–13 Hz)

In healthy individuals, background activity, assessed at rest with eyes closed, in occipital leads consists mainly of regular alpha rhythm (8–13 Hz), characterized by the highest power among all bands and easy to recognize in qualitative assessment (11, 58). Evaluation of background activity is one of the most important features analyzed in electroencephalography (59). Reduction in alpha wave proportion in background activity has been described in literature since the 1970s (7). Quantitative EEG analysis (qEEG) allows for precise assessment of alpha wave proportion in background activity (9). The 2008 review summarized contemporary knowledge, indicating reduced alpha wave dominance in patients with schizophrenia. Yeum and Kang (2018) conducted a study evaluating alpha wave characteristics in qEEG in 31 patients with schizophrenia compared to an age- and sex-matched control group (n=31) (37). Peak frequency, power, and alpha wave coherence were analyzed. Results showed significantly lower alpha wave peak frequency in the occipital region (Oz) in patients with schizophrenia compared to the control group. No differences in absolute or relative alpha wave power were found. Nakhnikian et al. (2024) investigated resting EEG characteristics in patients with schizophrenia compared to healthy controls, using principal component analysis (PCA) to decompose the power spectrum into components independent of *a priori* assumed frequency bands (41). They demonstrated decreased alpha peak frequency in patients with schizophrenia compared to the control group. Furthermore, they showed that the decrease in alpha peak frequency results from the presence of a unique spectral component in the theta/alpha range (6–9 Hz), characteristic only for patients with schizophrenia. The source distribution of this unique theta/alpha component involved mainly prefrontal and parahippocampal areas. Importantly, this finding provides a mechanistic explanation for the commonly observed reduction in alpha peak frequency in schizophrenia, suggesting it may not simply represent a reduction in Peak Alpha Frequency but rather the emergence of a pathological oscillation. Begić et al. (2011) in a study including 30 patients with schizophrenia, 30 patients with depression, and 30 healthy individuals showed decreased absolute alpha wave power in regions Fp1, Fp2, F3, F4, F7, T3, and T4 (34). In occipital regions, no decreased alpha activity was observed. Fuggetta et al. (2014) investigated whether quantitative EEG could serve as a biomarker of predisposition to psychosis development in individuals with schizotypal traits (60). They compared qEEG data in 16 individuals with low and 16 individuals with high levels of schizotypal traits. The study showed that individuals with high levels of schizotypal traits exhibited lower oscillation frequencies in the alpha band compared to individuals with low levels of schizotypy. Murphy and Öngür demonstrated that patients with first-episode psychosis had significantly lower peak alpha frequency during resting-state EEG compared with healthy controls, without concomitant differences in alpha power. This slowing was most prominent over central and posterior regions, suggesting that alpha rhythm dysregulation may already be present at the early stage of psychotic illness (44). Ramsay et al. (2021) reported lower individual alpha peak frequency (IAPF) during eyes-closed resting-state EEG in patients with schizophrenia compared to healthy controls, with reduced IAPF observed across all electrode sites (51). Ranlund et al. (2014) tested whether resting-state quantitative EEG could constitute an endophenotype of psychosis (35). They compared qEEG activity in four frequency bands in five groups: patients with chronic psychosis, patients with first-episode psychosis, individuals at risk of developing psychosis, healthy relatives of patients, and healthy control individuals. The study showed no significant differences in alpha wave amplitude between any of the studied groups. This result suggests that changes in resting alpha activity are not associated with genetic predisposition to psychosis. Kim et al. (2015) evaluated the utility of quantitative EEG in diagnosing pharmacologically untreated patients with schizophrenia (36). A study comparing qEEG characteristics of 90 patients with schizophrenia with 90 healthy control individuals at rest showed a significant decrease in alpha2 wave activity, particularly in fronto-central brain regions, in patients with schizophrenia. In the alpha1 band and beta band, no significant differences between groups were observed. Mitra et al. (2017) in a small 192-channel resting-state EEG study, patients with schizophrenia showed a significantly lower alpha-to-theta ratio (ATR) over the left fronto-temporal region compared with healthy controls. This finding suggests a localized imbalance of low-frequency oscillatory activity in schizophrenia (49). Renaldi et al. (2019) showed no significant differences in absolute alpha wave power between patients with first-episode psychosis and the control group (38). In contrast to studies reporting reduced alpha power or slowed peak alpha frequency, a large multisite resting-state EEG study found increased slow and fast alpha activity in patients with schizophrenia compared to healthy controls, likely reflecting differences in analytical approach, including the use of data-driven spectral decomposition (52). The lack of differences in the alpha band between patients with first-episode psychosis and the control group suggests that decreased alpha wave power may not be a characteristic feature of early-stage psychosis but may occur later in the disease course or be associated with other factors such as chronicity or long-term medication effects.

### 3.4 Beta frequency (13–30 Hz)

Kim et al. (2015) found no significant differences in beta activity between patients with schizophrenia and the control group (36). However, Begić et al. (2011) showed increased absolute beta wave power in patients with schizophrenia compared to the control group, particularly in frontal and temporal regions (34). Contradictory results regarding beta activity may result from methodological differences between studies, including different beta band definitions, different analysis methods (absolute vs. relative power), and differences in studied population characteristics (e.g., disease duration, medication use). Cecchi et al. (2023) in the resting-state EEG component showed reduced relative beta1 and beta2 power, and an increased theta/beta ratio in schizophrenia patients compared with controls (50).

The limited data and inconsistent findings suggest that beta band alterations in schizophrenia require further investigation with standardized methodologies.

### 3.5 Gamma frequency (>30 Hz)

Yadav et al. (2021) studied gamma wave spectral power at rest in patients with first-episode psychosis without prior neuroleptic treatment (39). The study compared gamma wave spectral power in 29 patients with first-episode psychosis with 30 healthy individuals. The study showed significantly higher gamma wave spectral power in the 31–50 Hz and 51–70 Hz bands in patients with first-episode psychosis compared to the control group. This effect was observed in most brain regions. Furthermore, disease duration was a predictor of gamma wave spectral power, showing negative correlation with power in frontal regions (right and left hemispheres) in the 31–50 Hz and 71–100 Hz bands, and in the temporal region (right hemisphere) in the 71–100 Hz band. These results suggest that changes in gamma activity may be characteristic of early psychosis stages and may change with disease progression. Tikka et al. investigated neuroleptic-naïve patients with first-episode schizophrenia using 192-channel eyes-closed resting-state EEG. Compared with healthy controls, patients showed significantly increased low-gamma power (30–50 Hz), particularly over the left frontal and left parietal regions (42). In a resting-state EEG study, patients with schizophrenia showed increased gamma-band power and reduced interhemispheric gamma coherence compared with healthy controls. These findings suggest that schizophrenia may be characterized by coexisting local gamma hyperactivity and impaired large-scale functional coupling (48). The limited number of studies examining gamma frequencies highlights this as an important area for future research.

A multicentered resting-state EEG study provided by Narayanan et al. supported these findings, demonstrating increased delta, theta, slow and fast alpha activity, as well as increased fronto-central slow beta activity in patients with schizophrenia compared to healthy controls (52).

## 4 Discussion

Research on schizophrenia biomarkers has been ongoing for decades but has not resulted in introducing any reliable marker into routine clinical practice. Boutros’s 2008 literature review demonstrated characteristic EEG spectral changes in patients with schizophrenia, independent of treatment received (5). However, the authors emphasized the lack of systematic research on translating findings into clinical practice, indicating an urgent need for methodology standardization and conducting multicenter studies. This systematic review, conducted according to PRISMA guidelines, aimed to assess research progress in qEEG 18 years after the Boutros review. Analysis of 19 original papers, selected from over 467 initially identified items, revealed a surprisingly small number of studies meeting inclusion criteria. In total, 1242 patients were included, comprising 981 individuals with schizophrenia and 261 with a first episode of psychosis, and these groups were compared with 1211 healthy control participants across the included studies. Despite the limited number of eligible publications, the cumulative sample size is considerable in the context of electrophysiological research, where individual studies typically involve relatively small cohorts. The inclusion of both patients with established schizophrenia and those experiencing a first episode of psychosis increases the representativeness of the analyzed population. The exclusion criteria consciously excluded evoked potential studies, task-based qEEG recordings, and studies using machine learning and artificial intelligence. Although these directions are promising, with studies achieving classification accuracies exceeding 70-90% for schizophrenia detection, their complex methodology, high costs, and limited reproducibility currently constitute barriers to clinical translation. This review focuses on classical qEEG parameters more readily applicable in clinical settings.

### 4.1 Summary of main findings

Analysis of papers published after 2008 showed that Boutros’s suggestions regarding methodology standardization have not been fully implemented, and studies remain characterized by significant methodological heterogeneity. Full meta-analysis cannot currently be performed due to differences in frequency band definitions, analysis parameters (absolute vs. relative power), electrode locations, and studied group characteristics (chronic schizophrenia vs. first-episode psychosis, treated vs. untreated). Based on analyzed studies, several recurring changes in EEG spectra can be distinguished in patients with schizophrenia compared to healthy individuals. In the delta band, most studies indicate increased activity in schizophrenia patients, particularly in anterior brain regions (34–36). However, Dominicus et al. (2023) showed no significant differences after correction for multiple testing, although differences were observed in narrow frequency bands (40). Increased delta activity may be associated with dysfunction of cortical and subcortical areas, consistent with the concept of neuronal integration disturbances in schizophrenia (34). In the theta band, studies by Begić and Kim showed increased activity in schizophrenia patients, particularly in anterior and central regions (34, 36). Ranlund et al. (2014) observed increased theta amplitude only in chronic psychosis patients, suggesting changes may relate to disease duration or long-term antipsychotic use (35). The innovative work by Nakhnikian et al. (2024) provides new insights, identifying a unique theta/alpha component (6–9 Hz) present only in schizophrenia patients (41). This suggests theta band abnormalities may represent a specific pathological oscillation rather than simply increased normal theta activity. Increased theta activity may reflect disturbances in cognitive functions and working memory (36). Changes in the alpha band appear more complex and inconclusive. Yeum and Kang (2018) and Nakhnikian et al. (2024) showed decreased alpha peak frequency, suggesting background activity slowing (37, 41). The mechanistic insights provided by Nakhnikian et al. demonstrate that alpha slowing results from the presence of the unique theta/alpha component (6–9 Hz), providing a novel explanation for this commonly observed phenomenon. The findings of Ramsay et al. suggests that alpha peak frequency may capture a functionally relevant aspect of resting-state dysfunction, as lower IAPF was linked to poorer visual attention and global cognition, although these associations extend beyond the core scope of the present review (51). Begić et al. (2011) observed decreased absolute alpha power in frontal and temporal regions, but not occipital regions (34). Kim et al. (2015) found decreased alpha2 activity, but not alpha1 (36). Importantly, studies of first-episode psychosis patients showed no significant differences in absolute alpha wave power between patients and controls (38, 61). This suggests decreased alpha wave power may not characterize early-stage psychosis but may occur later in disease course or be associated with other factors. Decreased alpha activity, particularly in posterior regions, may indicate disturbances in sensory processing and inhibition mechanisms (37). However, a large multicentered study reported increased slow and fast alpha activity in schizophrenia, suggesting that alpha-band findings may be highly dependent on the analytical approach, particularly when data-driven spectral decomposition methods are used (52). Recent studies have increasingly examined alpha peak frequency in the context of non-diagnostic, dimensional features, such as aggression, rather than its role as a diagnostic biomarker. In such frameworks, group-level differences are less consistently observed, which may reflect both clinical heterogeneity and differences in study objectives (62). Recent studies have also explored alpha oscillations in schizophrenia within interventional frameworks, for example by examining whether modulation of alpha-band activity through non-invasive stimulation affects symptoms and network-level measures. However, such studies do not provide diagnostic resting-state qEEG comparisons with healthy controls and therefore fall outside the core scope of the present review (63). In the beta band, results are contradictory. Begić et al. (2011) observed increased beta power in schizophrenia patients, while Kim et al. (2015) found no significant differences (34, 36). Increased beta activity may be associated with disturbances in excitation-inhibition balance (34). In the gamma band (>30 Hz), Yadav et al. (2021) showed increased power in first-episode psychosis patients, suggesting disturbances may be present in early disease stages (39). This is particularly important because gamma oscillations are associated with sensory information integration and higher-order cognitive functions disturbed in schizophrenia (39).

### 4.2 Differential patterns: first-episode psychosis vs. chronic schizophrenia

Comparative analysis presented in **Table 3** reveals important differences in qEEG changes between first-episode psychosis and chronic schizophrenia, providing crucial insights into disease progression. The systematic comparison demonstrates that qEEG alterations follow a clear developmental trajectory, with subtle or absent changes in first-episode psychosis evolving into consistent and pronounced abnormalities in chronic schizophrenia. This progressive pattern is most evident in delta and theta frequency bands, where first-episode patients show either no significant differences (35, 61) or mixed findings, while chronic schizophrenia patients consistently demonstrate increased activity across multiple studies (34–36). The alpha band findings are particularly informative, as the characteristic reduction in peak frequency and power appears predominantly a feature of chronic schizophrenia rather than early-stage illness (37). The discovery of the unique theta/alpha component by Nakhnikian et al. (2024) exclusively in chronic patients further supports the concept of progressive neurophysiological deterioration (41). Interestingly, gamma band alterations show the opposite pattern, with increased power observed in first-episode psychosis, suggesting different frequency bands may reflect distinct pathophysiological processes at various disease stages (39). This differentiation may result from several factors. Changes in qEEG may intensify with disease duration, reflecting progressive structural and functional brain changes (20). This suggests occurrence of a very long prodromal period in which neuropathological and neurophysiological changes occur before manifestation of full-blown schizophrenia. Therefore, the search for biomarkers enabling differentiation of healthy individuals from those in prodromal stages is particularly important. Another important aspect is long-term antipsychotic use, which may affect brain electrical activity. Studies evaluating medication effects on qEEG recording are needed, both for treatment response and prediction of antipsychotic treatment outcomes (30). Furthermore, first-episode psychosis may include various disorders that ultimately develop into schizophrenia or other psychotic disorders, which may lead to greater variability in qEEG results (38). This observation emphasizes the need for longitudinal studies tracking qEEG changes from first-episode psychosis through subsequent disease years, which would allow better understanding of neurophysiological change dynamics in schizophrenia.

### 4.3 Methodological challenges and limitations

The review revealed numerous methodological challenges hindering the comparison between studies and drawing clear conclusions. First, non-uniform frequency band definitions-different studies use different ranges for individual bands (e.g., delta: 0.5–4 Hz vs. 1.95-3.90 Hz)-hinder direct result comparison (11). Another aspect is that studies use different qEEG parameters, such as absolute power, relative power, peak frequency, and coherence, which complicates result analysis and statistical synthesis. We also noted varied reporting of obtained results. Some studies focus on individual electrodes, others on regions (e.g., anterior, central, posterior), and still others on the entire head surface. A very important element is the lack of standardization in artifact identification and removal, which may lead to differences in analyzed EEG signal quality (3). Furthermore, most analyzed studies included relatively small sample sizes (20–90 participants), which may limit statistical power and generalizability of findings. Authors presented different approaches to statistical analysis, particularly differences in multiple testing correction methods, which may lead to different conclusions about the statistical significance of observed changes. This is exemplified by the Dominicus et al. (2023) study, where significant differences disappeared after correction for multiple comparisons, yet were evident in narrow frequency band analyses. An important consideration is the clinical significance of observed changes. While many studies report statistically significant differences in qEEG parameters, the clinical utility of these findings remains unclear. For instance, a 2 Hz reduction in alpha peak frequency may be statistically significant, but whether this represents a clinically meaningful change that could inform diagnosis or treatment decisions requires further investigation. This variability may be further amplified by differences in analytical strategies, as some studies rely on conventional band-limited power measures, whereas others apply data-driven approaches such as independent component analysis, which may yield partially divergent spectral patterns (52). These methodological challenges are consistent with Boutros’s 2008 observations and emphasize the need for standardization of qEEG research methodology in schizophrenia, which could accelerate progress toward clinical application (5).

### 4.4 Potential clinical applications

Despite methodological challenges, the review identifies several potential clinical applications of qEEG in schizophrenia. Characteristic qEEG patterns, such as increased delta and theta activity and decreased alpha peak frequency, could help differentiate schizophrenia from other psychiatric and neurological disorders, such as ADHD, anxiety disorders or dementia, though this area requires further research (64). One study suggests that qEEG changes may be present in individuals with high levels of schizotypal traits, which could help identify individuals at increased risk of psychosis development (60). Distinguishing differences in qEEG profiles in patients with confirmed schizophrenia may reflect different disease subtypes, which could lead to a more personalized therapeutic approach (40). Changes in qEEG may reflect disease pathophysiology progression, which could be useful in long-term monitoring of disease course and treatment response (30). Moreover, qEEG could serve as an objective biomarker of treatment response, although this aspect extends beyond this review’s scope (20). The innovative findings from Nakhnikian et al. (2024) regarding the unique theta/alpha component (6–9 Hz) in schizophrenia provide a particularly potentially promising avenue for future biomarker development (41). Although source-localization methods such as LORETA were not included in the present review, it is worth noting that recent sLORETA studies suggest that resting-state EEG abnormalities may have clinical relevance beyond scalp-level spectral measures. These findings include potential associations with later transition to psychosis. For example, a recent sLORETA study in drug-free individuals at risk of psychosis reported increased β1 current source density in the left middle frontal gyrus in those who later developed psychosis. This supports the broader clinical relevance of resting-state EEG-based biomarkers (65).

## 5 Conclusions

This systematic review, covering studies published after 2008, confirms the existence of characteristic changes in qEEG spectra in patients with schizophrenia, particularly increased delta and theta wave activity and decreased alpha wave peak frequency. However, these changes are more pronounced in patients with chronic schizophrenia than in patients with first-episode psychosis, which may indicate their relationship with disease progression or long-term treatment effects. The unique theta/alpha component (6–9 Hz) identified by Nakhnikian et al. (2024) represents a novel mechanistic insight into the neurophysiological basis of reduction in peak alpha frequency in schizophrenia and may serve as a more specific potential biomarker warranting further investigation (41). Methodological heterogeneity of qEEG studies in schizophrenia remains a significant challenge 18 years after the Boutros review. This limits the possibility of conducting meta-analysis and formulating definitive conclusions. Differences in frequency band definitions, analyzed parameters, electrode locations, and statistical methods continue to hinder result comparison between studies. Despite these limitations, the review identifies several promising research directions including narrow frequency band analysis, data-driven approaches, advanced signal analysis methods, longitudinal studies, multimodal biomarker integration, and multicenter studies with standardized protocols and large samples. In summary, resting-state qEEG remains a promising potential biomarker of schizophrenia, however, in light of the current state of evidence and its limited clinical applicability, it cannot yet be regarded as a diagnostic biomarker of the disorder (66–68). Nevertheless, its implementation in routine clinical practice requires further investigation based on standardized protocols and advanced data analysis methods (64). Development in this field could contribute to more objective schizophrenia diagnosis, treatment personalization, and better understanding of the pathophysiology of this complex disorder.

Given the rapid advancement of artificial intelligence, it is highly likely that quantitative EEG (qEEG) techniques will increasingly require integration with machine learning approaches to fully exploit their diagnostic and predictive potential (69). This trend is already reflected in the growing number of scientific publications focusing on the application of supervised and unsupervised learning algorithms to electrophysiological data (70) Machine learning methods offer enhanced capabilities in pattern recognition, multidimensional data integration, and classification, which may improve the sensitivity and specificity of qEEG-based biomarkers (71, 72). However, due to current technical, infrastructural, and financial constraints, the routine implementation of such advanced analytical frameworks in everyday clinical practice remains limited. Nevertheless, the authors emphasize the importance of continued monitoring, critical appraisal, and systematic evaluation of these emerging methodologies in the field of quantitative electroencephalography, as their clinical applicability is likely to expand in the future (73, 74).

## Data Availability

The original contributions presented in the study are included in the article/supplementary material, further inquiries can be directed to the corresponding author/s.
